# OCT-Based Retinal Vasculature Analysis: Age, Sex, and Body Mass Index Associations in the Nagahama Study, a Large Japanese Cohort

**DOI:** 10.1016/j.xops.2025.100740

**Published:** 2025-02-12

**Authors:** Ran Xiang, Yuki Muraoka, Takahiro Kogo, Yuki Mori, Masahiro Miyake, Yu Hidaka, Satoshi Morita, Yasuharu Tabara, Fumihiko Matsuda, Akitaka Tsujikawa

**Affiliations:** 1 Department of Ophthalmology and Visual Sciences, Kyoto University Graduate School of Medicine, Kyoto, Japan; 2 Department of Biomedical Statistics and Bioinformatics, Kyoto University Graduate School of Medicine, Kyoto, Japan; 3 Institute for Advancement of Clinical and Translational Science, Kyoto University Hospital, Kyoto, Japan; 4 Graduate School of Public Health, Shizuoka Graduate University of Public Health, Shizuoka, Japan; 5 Center for Genomic Medicine, Kyoto University Graduate School of Medicine, Kyoto, Japan

**Keywords:** OCT, Inner and outer diameter, Vessel wall thickness and reflectivity, Demographic and anthropometric factors, Nagahama Study

## Abstract

**Objective:**

This study aimed to evaluate retinal vessel structure using OCT, which enables high-resolution imaging for detailed vascular assessment. We investigated how age, sex, and body mass index (BMI) influence the OCT-measured parameters, including outer and inner diameters (ODs and IDs, respectively), wall thickness, and wall reflectivity—parameters that are challenging to assess using color fundus photography.

**Design:**

A cross-sectional retrospective study.

**Participants:**

The study included 6981 participants in the Nagahama Study, with 6981 eyes being assessed.

**Methods:**

OCT B-scan images centered on the optic disc were obtained. For each participant, mean values of the ODs and IDs, wall thickness, and wall reflectivity of the 4 largest arteries and veins were measured.

**Main Outcome Measures:**

Associations of retinal vessel parameters with age, sex, and BMI were evaluated. The reliability of OCT-measured parameters was assessed using intraclass correlation coefficients. Multivariable linear regression adjusted for intraocular pressure and axial length was used to investigate the associations with demographic and anthropometric factors.

**Results:**

Intraclass correlation coefficients for retinal vessel parameters demonstrated good-to-excellent reliability (0.767–0.957, *P* < 0.001). Compared with those of veins, arterial diameters were smaller, and arterial wall thickness and reflectivity were greater. Multivariable analysis revealed a U-shaped association between age and arterial diameter. Participants aged ≥60 years had significantly larger diameters than those aged 30 to 40 years and those in their 50s. Venous diameter decreased linearly with age. The arterial wall thickness and reflectivity increased with age. Women exhibited larger arterial diameters than men. Body mass index was negatively associated with the arterial diameter and positively associated with the venous diameter and arterial wall thickness.

**Conclusions:**

OCT enabled detailed evaluation of retinal vessel structure, allowing for the measurement of parameters that are challenging to assess by fundus photography, such as IDs and ODs, wall thickness, and wall reflectivity. This study, conducted in a large Japanese cohort, demonstrated significant associations between these OCT-measured retinal vascular parameters and age, sex, and BMI. These findings support the potential of OCT as a valuable tool for objective, in-depth assessment of retinal vascular health and its relationships with demographic and anthropometric factors.

**Financial Disclosure(s):**

Proprietary or commercial disclosure may be found in the Footnotes and Disclosures at the end of this article.

The retinal vasculature is one of the few vascular systems that can be visualized directly and noninvasively through transparent ocular media. This unique accessibility enables the detection of systemic vascular changes, such as arteriolar narrowing and increased vascular reflex, which have traditionally been used to assess arteriosclerosis and hypertensive changes through fundus photography.^1^^,^^2^ However, these evaluations are often subjective and susceptible to interobserver variability.

Quantitative systems for evaluating retinal vessel caliber using fundus photography have been developed to enhance objectivity.^3^, ^4^, ^5^, ^6^ These standardized approaches have revealed significant associations between retinal vessel caliber and systemic conditions, such as hypertension and cardiovascular disease.^7^, ^8^, ^9^ However, the inability of fundus photography to resolve vessel wall structures limits its ability to differentiate between inner and outer vessel diameters or measure wall thickness. In contrast, systemic vascular assessments often include parameters, such as wall-to-lumen ratios, which offer valuable insights into arterial stiffness and atherosclerosis.^10^^,^^11^

OCT has significantly advanced retinal imaging by providing high-resolution, noninvasive, cross-sectional images, enabling the quantitative assessment of vessel wall thickness and reflectivity. This enhanced resolution allows for a more detailed evaluation of retinal vascular health and its relationship with demographic and anthropometric factors, thus contributing to a more comprehensive understanding of vascular conditions.^12^

We aimed to develop a semiautomated system to measure retinal vascular parameters (outer and inner diameters [ODs and IDs, respectively], wall thickness, and wall reflectivity) using OCT B-scan images, focusing on demographic and anthropometric factors, such as age, sex, and body mass index (BMI), as these are stable and consistently measurable variables that are ideal for alidating the system's accuracy and reliability. We aimed to examine how these factors influence OCT-measured retinal vessel parameters and to investigate the potential utility of OCT in assessing retinal vascular health.

## Methods

### Study Participants and Data Collection

Approval was obtained from the Institutional Review Board of the Kyoto University Graduate School of Medicine and the Nagahama Municipal Review Board. The study adhered to the tenets of the Declaration of Helsinki. Written informed consent was obtained from all participants before any procedures or examinations.

We analyzed data collected from the second visit to the Nagahama Prospective Cohort for Comprehensive Human Bioscience (Nagahama Study, Japan) between 2013 and 2016. The Nagahama Study is a large, community-based cohort study designed to investigate environmental and genetic factors contributing to various health conditions in the Japanese population; it has been widely used for systemic and ocular health researches.^13^, ^14^, ^15^ We examined data from 9850 individuals aged 34 to 80 years who underwent OCT B-scans of a 3.45-mm diameter circular cross-section centered on the optic disc using spectral-domain OCT (RS-3000 Advance; NIDEK).

Exclusion criteria included a history of any ocular surgery (except for cataract surgery), high myopia (axial length [AL] >26 mm), hyperopia (AL <21 mm), a history of significant retinal diseases, such as glaucoma, retinal vascular diseases, macular pathologic changes (e.g., age-related macular degeneration, epiretinal membrane), and retinal detachment. We also excluded participants with poor-quality OCT images, which were defined as those with a signal strength index <5 or images compromised by factors such as eye movement or media opacities that impaired vascular assessment, as judged by ≥1 evaluators. Participants who were pregnant or had data loss were further excluded.

Ophthalmological examinations included measurements of spherical equivalent (ARK-530A; NIDEK), intraocular pressure (IOP) (TX-20P; Canon), AL (IOL Master; Carl Zeiss Meditec, Inc.), color fundus photography (CR-DG10; Canon), and spectral-domain OCT. The RS-3000 OCT system specifications included a scanning speed of 53 000 A-scans/second, an optical resolution of 20 μm (X-Y) and 7 μm (Z), a digital resolution of 3 μm (X-Y) and 4 μm (Z), and a scanning depth of 2.1 mm.

Body mass index was calculated as weight (kg) divided by height squared (m^2^) and categorized according to the World Health Organization criteria as follows: underweight (<18.5 kg/m^2^); normal weight (18.5–24.9 kg/m^2^), which is further subdivided into lower normal weight (18.5–21.9 kg/m^2^) and upper normal weight (22.0–24.9 kg/m^2^); and overweight (≥25 kg/m^2^). Medical history was assessed using a questionnaire and examination.

### Measurement and Calculation of Vessel Parameters

**Figure 1:**
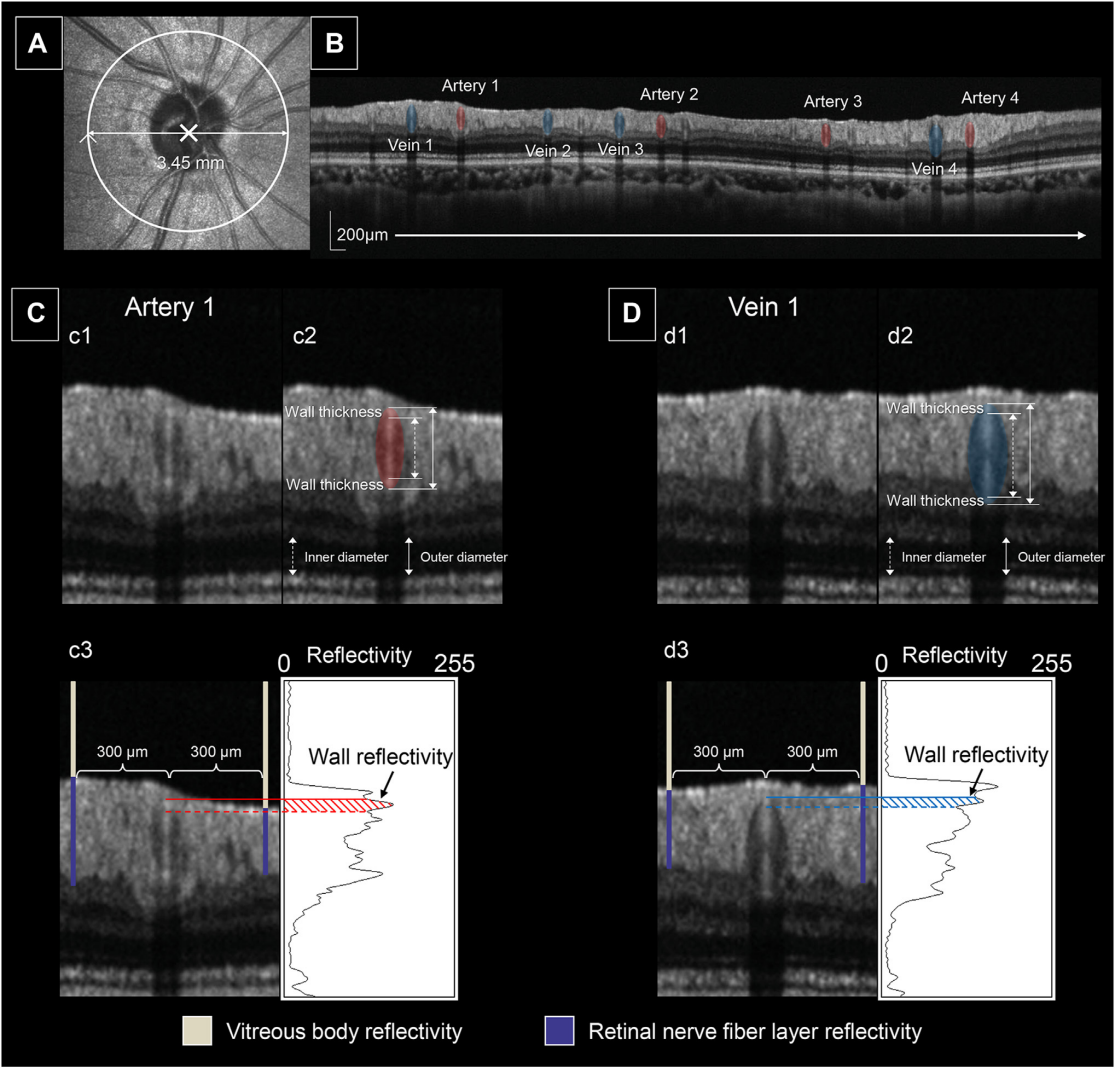
OCT-based measurement of retinal vessel parameters. **A,** Scanning laser ophthalmoscopy image. The white circular arrow represents the circular OCT scan around the optic disc, indicating the location of the scan and the scan direction used to acquire cross-sectional images of retinal vessels. **B,** Circular OCT image around the optic disc. Cross-sectional images of retinal arteries (red) and veins (blue) are shown. The 4 largest arteries and veins were selected for vessel parameter measurements. **C,** Retinal arterial cross-sections. **D,** Venous cross-sections: c1, d1. OCT B-scans of vessels: c2, d2. The *red oval* outlines the arterial cross-section and the *blue oval* outlines the venous cross section, respectively. The outer and inner borders of the vessels are defined. The distance between the outer borders is considered the OD, and the distance between the inner borders is considered the ID. Half the difference between these 2 distances is defined as the wall thickness: c3, d3. Solid and dashed lines represent the outer and inner borders of the superficial vessel walls, respectively. The average wall reflectivity is measured along a vertical line passing through the center of the vessel. The average reflectivity of the vitreous body and RNFL is referenced from lines located 300 μm away from the central line of the vessel. Using the following formula, the reflectivity level is set to zero in the vitreous body and 100 in the RNFL: the vessel parameters of the 4 largest arteries and veins are averaged for each participant. ID = inner diameter; OD = outer diameter; RNFL = retinal nerve fiber layer.

Retinal vessel parameters were measured using semiautomatic measurement software (NAVIS-EX, NIDEK) on OCT B-scan images (Fig 1). Retinal vessel sections were analyzed within a 3.45-mm-diameter circular cross-section centered on the optic disc. The software initially automatically identified the locations of retinal vessels and differentiated arteries from veins based on scanning laser ophthalmoscopy images acquired simultaneously with the OCT B-scan. The accuracy of vessel detection was verified by cross-checking with color fundus photographs. The central lines of these vessel locations were then mapped onto the corresponding vessels in the OCT B-scan for further evaluation. The software automatically detected the centers of the vessels and borders of the vessel walls in the OCT B-scan images. After these detections, the examiner refined the vessel centers and wall borders manually, based on the reflectivity patterns in the OCT B-scan images. After data export, the 4 largest arteries and veins, determined by their ODs, were selected for analysis, and the mean values were calculated for each participant.

#### Outer Diameters and IDs (μm)

The mean ODs and IDs of the arteries and veins were calculated using the following formula:

$$\text{OD}=\frac{OD_{1\text{st}}+{\text{OD}}_{2\text{nd}}+{\text{OD}}_{3\text{rd}}+{\text{OD}}_{4\text{th}}}{4}$$

$$\text{ID}=\frac{{\text{ID}}_{1\text{st}}+{\text{ID}}_{2\text{nd}}+{\text{ID}}_{3\text{rd}}+{\text{ID}}_{4\text{th}}}{4}$$

#### Wall Thickness (μm)

The mean wall thicknesses of the arteries and veins were calculated as the average of half the difference between the ODs and IDs of the 4 largest vessels using the following formula:

$$\text{Wall}\;\text{thickness}=\frac{\frac{{\text{OD}}_{1\text{st}}-{\text{ID}}_{1\text{st}}}{2}+\frac{{\text{OD}}_{2\text{nd}}-ID_{2\text{nd}}}{2}+\frac{{\text{OD}}_{3\text{rd}}-ID_{3\text{rd}}}{2}+\frac{OD_{4\text{th}}-ID_{4\text{th}}}{2}}{4}$$

#### Wall Reflectivity (Arbitrary Units)

The wall reflectivity was calculated for the same arteries and veins used for the vessel diameter and wall thickness measurements. Specifically, only the reflectivity of the superficial vessel wall was evaluated because the reflectivity of the deeper wall may be influenced by the blockage of intravascular red blood cells. The reflectivity of the vitreous body and retinal nerve fiber layer (RNFL) was used as a reference based on previously reported methods to account for individual variations in OCT reflectivity.^16^^,^^17^ The formula used was as follows:

$$\text{Wall}\;\text{reflectivity}=\frac{\left(\text{Raw}\;\text{wall}\;\text{reflectivity}-\text{Vitreous}\;\text{body}\;\text{reflectivity}\right)}{\left(\text{RNFL}\;\text{reflectivity}-\text{Vitreous}\;\text{body}\;\text{reflectivity}\right)}\times 100$$

For each vessel, a perpendicular line was drawn through the center, with 2 additional lines placed 300 μm on either side. Raw wall reflectivity was measured and defined as the average reflectivity along the center line. The reflectivity of the vitreous layer and RNFL was measured along the 2 surrounding lines and averaged. The wall reflectivity for each participant was calculated as the mean value of the 4 largest arteries and veins. All calculations were performed using integrated software.

The intraobserver reproducibility was evaluated by having the same examiner to remeasure 100 participants on 2 separate occasions to confirm the reliability of the OCT measurements. Interobserver reproducibility was assessed by 2 different examiners using the same 50 participants.

### Statistical Analysis

Retinal vessel parameters, including ODs and IDs, wall thickness, and reflectivity, were treated as continuous variables and are presented as mean ± standard deviation (or with 95% confidence intervals [CIs]). Categorical data were analyzed as binary or categorical variables and presented as frequencies and proportions.

Intraclass correlation coefficients (ICCs) were calculated for intraobserver and interobserver reproducibility to assess the reliability of OCT-based retinal vessel measurements.

Multivariable linear regression analyses were conducted to assess the independent associations between retinal vessel parameters and demographic and anthropometric factors (age, sex, and BMI). In these models, retinal vessel parameters (ODs, IDs, wall thickness, and reflectivity) served as dependent variables, whereas age, sex, BMI, AL, and IOP were independent variables. Adjustments were made for each factor, including age, sex, BMI, AL, and IOP, to control for demographic and ocular factors that could influence retinal vessel parameters.^18^^,^^19^ Age was categorized into 3 groups: 30s to 40s, 50s (reference group), and 60s to 80s. Sex was categorized as male (reference group) or female, and BMI was categorized into underweight, normal weight (reference group), and overweight.

All statistical analyses were performed using IBM SPSS Statistics software (version 27.0; IBM Co.). Graphs were generated using GraphPad Prism (version 10, GraphPad Software). *P* values <0.05 were considered statistically significant.

## Results

**Figure 2:**
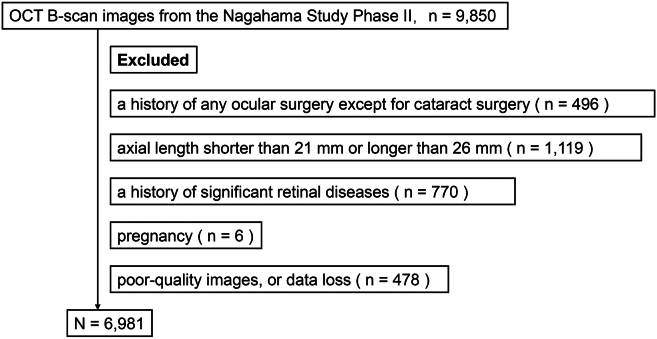
Flow chart of the exclusion criteria for study eyes. To ensure data quality and avoid confounding factors, we excluded participants with a history of any ocular surgery (except cataract surgery, n = 496), axial length <21 mm or >26 mm (n = 1119), or a history of significant retinal diseases such as glaucoma, retinal vascular diseases, macular pathologic changes (e.g., age-related macular degeneration and epiretinal membrane), and retinal detachment (n = 770). Pregnant participants (n = 6) and those with poor-quality images or data loss (n = 478) were also excluded. After applying these exclusion criteria, 6981 participants remained for the final analysis.

After the criteria described in the Methods section, we analyzed data from 6981 participants (Fig 2). The participants had a mean age of 57.6 ± 12.1 years, with a mean BMI of 22.20 ± 3.31 kg/m^2^, a mean AL of 23.78 ± 1.01 mm, and a mean IOP of 14.23 ± 2.47 mmHg (Table 1).

**Table 1:** Participant Characteristics (N = 6981)

Characteristics	Summary Statistics
Sex, n (%)	
Male	2129 (30.5%)
Female	4852 (69.5%)
Age, yrs (95% CI) (range)	57.6 ± 12.1 (57.4–57.9) (34.2–80.5)
30s, n (%)	631 (9.0%)
40s, n (%)	1520 (21.8%)
50s, n (%)	1466 (21.0%)
60s, n (%)	2079 (29.8%)
70s, n (%)	1285 (18.4%)
BMI, kg/m^2^ (range)	22.20 ± 3.31 (12.9–45.1)
Underweight (BMI <18.5 kg/m^2^), n (%)	719 (10.3%)
Normal weight (BMI 18.5–24.9 kg/m^2^)	
18.5–21.9 kg/m2, n (%)	2863 (41.0%)
22.0–24.9 kg/m^2^, n (%)	2154 (30.9%)
Overweight (BMI ≥25 kg/m^2^), n (%)	1245 (17.8%)
Axial length, mm (95% CI)	23.78 ± 1.01 (23.76–23.81)
Intraocular pressure, mmHg (95% CI)	14.23 ± 2.47 (14.16–14.29)

BMI = body mass index; CI = confidence interval.

Data are presented as the mean ± standard deviation unless otherwise indicated.

The reliability of OCT-based retinal vessel measurements was evaluated using ICCs, which demonstrated good-to-excellent reproducibility (Table 2). For arterial parameters, the ICCs ranged from 0.767 to 0.841 for intraobserver reproducibility and from 0.815 to 0.917 for interobserver reproducibility. For venous parameters, the ICC ranged from 0.824 to 0.924 for intraobserver reproducibility and from 0.816 to 0.957 for interobserver reproducibility.

**Table 2:** Intraclass Correlation Coefficient for OCT-Measured Retinal Vessel Parameters

Retinal Vessel Parameters	Intraobserver Reproducibility (n = 100)			Interobserver Reproducibility (n = 50)		
	Correlation Coefficient	95% CI	*P* Value	Correlation Coefficient	95% CI	*P* Value
Artery						
Outer diameter	0.818	0.740–0.873	<0.001	0.826	0.711–0.897	<0.001
Inner diameter	0.767	0.673–0.837	<0.001	0.815	0.695–0.891	<0.001
Wall reflectivity	0.841	0.773–0.890	<0.001	0.917	0.855–0.951	<0.001
Vein						
Outer diameter	0.867	0.808–0.908	<0.001	0.838	0.731–0.905	<0.001
Inner diameter	0.824	0.749–0.878	<0.001	0.816	0.696–0.891	<0.001
Wall reflectivity	0.924	0.888–0.948	<0.001	0.957	0.925–0.975	<0.001

CI = confidence interval.

Arterial diameters (OD: 117.5 μm, ID: 89.4 μm) were significantly smaller than venous diameters (OD: 138.5 μm, ID: 113.2 μm; *P* < 0.001 for both). Arterial wall thickness (14.0 μm) and reflectivity (137.6) were significantly greater than venous wall thickness (12.6 μm) and reflectivity (120.7) (*P* < 0.001 for both, Table 3).

**Table 3:** Comparison of Retinal Vessel Parameters between Arteries and Veins

Retinal Vessel Parameters	Artery Mean ± SD, (95% CI)	Vein Mean ± SD, (95% CI)	*P* Value
Outer diameter, μm	117.5 ± 8.8 (117.2–117.7)	138.5 ± 11.0 (138.3–138.8)	<0.001
Inner diameter, μm	89.4 ± 8.1 (89.2–89.6)	113.2 ± 10.3 (113.0–113.5)	<0.001
Wall thickness, μm	14.0 ± 1.4 (14.0–14.1)	12.6 ± 1.4 (12.6–12.7)	<0.001
Wall reflectivity, AU	137.6 ± 14.6 (137.2–137.9)	120.7 ± 10.9 (120.5–121.0)	<0.001

AU = arbitrary units; CI = confidence interval; SD = standard deviation.

Data are presented as the mean ± standard deviation.

### Association between Demographic and Anthropometric Factors and Vessel Parameters

**Figure 3:**
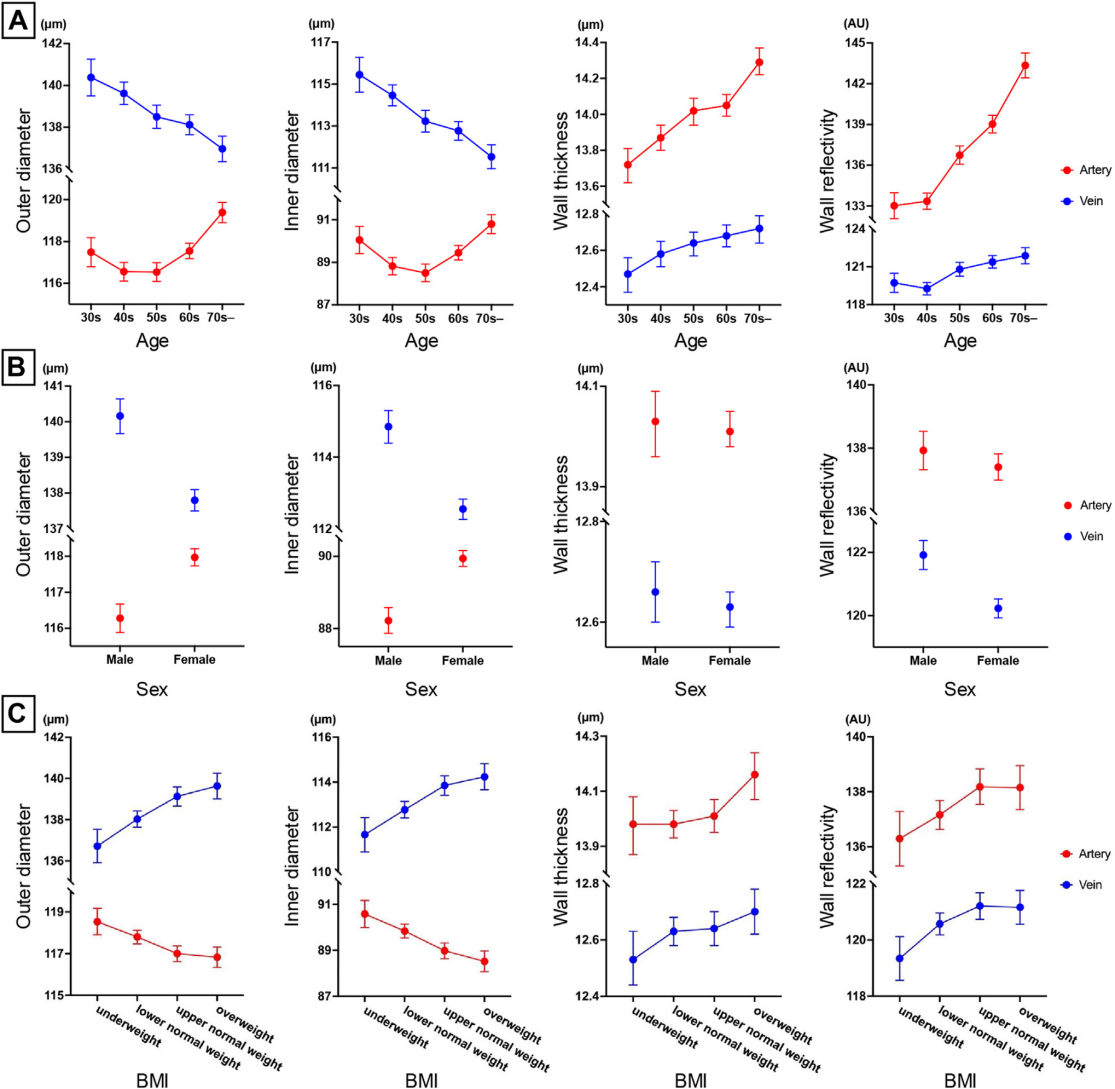
Retinal vessel parameters stratified by demographic and anthropometric factors. This figure shows line graphs with error bars (mean with 95% confidence intervals) depicting the distribution of retinal vessel parameters (arterial and venous diameters, wall thickness, and reflectivity) stratified by age (**A**), sex (**B**), and BMI (**C**). Arteries are shown in red, and veins are shown in blue. BMI = body mass index.

Figure 3 shows the means and 95% CIs for the vessel parameters according to age, sex, and BMI. Arterial diameters showed a U-shaped appearance with age, decreasing around the 50s and increasing in those in their 60s and older.

In the multivariable analysis (Table 4), the outer arterial diameters were significantly larger in participants aged ≥60 years compared with those in their 50s (β = 1.923, 95% CI: 1.281–2.565, *P* < 0.001). Inner arterial diameters were also significantly larger in participants in both the 30s to 40s (β = 0.847, 95% CI: 0.207–1.486, *P* = 0.009) and the 60s to 80s (β = 1.684, 95% CI: 1.091–2.276, *P* < 0.001) compared with those in their 50s.

**Table 4:** Multivariable Analysis of Associations between Retinal Vessel Parameters and Demographic and Anthropometric Factors

Artery												
	Outer Diameter			Inner Diameter			Wall Thickness			Wall Reflectivity		
	β	95% CI for β	*P* Value	β	95% CI for β	*P* Value	β	95% CI for β	*P* Value	β	95% CI for β	*P* Value
Age, yrs												
30s–40s	0.597	−0.095 to 1.290	0.091	0.847	0.207–1.486	0.009	−0.125	−0.236 to −0.014	0.028	−4.052	−5.207 to −2.897	<0.001
50s (ref.)	ref.	n.a.	n.a.	ref.	n.a.	n.a.	ref.	n.a.	n.a.	ref.	n.a.	n.a.
60s–80s	1.923	1.281–2.565	<0.001	1.684	1.091–2.276	<0.001	0.120	0.017–0.223	0.022	4.104	3.033–5.174	<0.001
Sex												
Male (ref.)	ref.	n.a.	n.a.	ref.	n.a.	n.a.	ref.	n.a.	n.a.	ref.	n.a.	n.a.
Female	1.749	1.235–2.263	<0.001	1.744	1.270–2.219	<0.001	0.002	−0.080 to 0.085	0.955	0.588	−0.269 to 1.445	0.178
BMI												
Underweight	0.910	0.158–1.661	0.018	0.775	0.082–1.469	0.028	0.067	−0.053 to 0.188	0.273	−0.634	−1.887 to 0.619	0.321
Normal weight (ref.)	ref.	n.a.	n.a.	ref.	n.a.	n.a.	ref.	n.a.	n.a.	ref.	n.a.	n.a.
Overweight	−0.295	−0.894 to 0.304	0.334	−0.686	−1.239 to −0.133	0.015	0.195	0.100–0.291	<0.001	0.308	−0.691 to 1.307	0.546

**Vein**												

Age, yrs												
30s–40s	2.068	1.204–2.931	<0.001	2.077	1.266–2.887	<0.001	−0.004	−0.112 to 0.103	0.936	−1.360	−2.228 to −0.492	0.002
50s (ref.)	ref.	n.a.	n.a.	ref.	n.a.	n.a.	ref.	n.a.	n.a.	ref.	n.a.	n.a.
60s–80s	−1.231	−2.031 to −0.431	0.003	−1.289	−2.040 to −0.538	0.001	0.029	−0.071 to 0.129	0.568	0.632	−0.172 to 1.436	0.124
Sex												
Male (ref.)	ref.	n.a.	n.a.	ref.	n.a.	n.a.	ref.	n.a.	n.a.	ref.	n.a.	n.a.
Female	−2.577	−3.218 to −1.937	<0.001	−2.503	−3.104 to −1.902	<0.001	−0.037	−0.117 to 0.043	0.363	−1.368	−2.012 to −0.725	<0.001
BMI												
Underweight	−1.777	−2.714 to −0.841	<0.001	−1.688	−2.567 to −0.809	<0.001	−0.045	−0.162 to 0.072	0.454	−0.903	−1.845 to 0.038	0.060
Normal weight (ref.)	ref.	n.a.	n.a.	ref.	n.a.	n.a.	ref.	n.a.	n.a.	ref.	n.a.	n.a.
Overweight	0.918	0.171–1.664	0.016	0.736	0.035–1.437	0.039	0.091	−0.003 to 0.184	0.057	−0.065	−0.816 to 0.685	0.865

β = coefficient beta; BMI = body mass index; CI = confidence interval; n.a. = not applicable; ref. = reference.

Multivariable analyses were conducted to evaluate the influence of age, sex, BMI, axial length, and intraocular pressure on OCT parameters.

Venous diameters decreased linearly with age, with significantly smaller ODs (β = −1.231, 95% CI: −2.031 to −0.431, *P* < 0.001) and IDs (β = −1.289, 95% CI: −2.040 to −0.538, *P* = 0.001) in participants aged ≥60 years compared with those in their 50s.

Arterial wall thickness increased with age, with significantly higher values observed in participants aged ≥60 years (β = 0.120, 95% CI: 0.017–0.223, *P* = 0.001). Arterial wall reflectivity also increased significantly with age (β = 4.104, 95% CI: 3.033–5.174, *P* < 0.001). Venous wall reflectivity increased with age, though less prominently than arterial reflectivity, particularly in those aged ≤60 years (β = −1.360, 95% CI: −2.228 to −0.492, *P* = 0.002).

### Sex Differences

Female individuals had significantly larger arterial diameters (OD: β = 1.749, 95% CI: 1.235–2.263, *P* < 0.001; ID: β = 1.744, 95% CI: 1.270–2.219, *P* < 0.001) and significantly smaller venous diameters (OD: β = −2.577, 95% CI: −3.218 to −1.937, *P* < 0.001; ID: β = −2.503, 95% CI: −3.104 to −1.902, *P* < 0.001), compared with men. Additionally, venous wall reflectivity was slightly higher in men (β = −1.368, 95% CI: −2.012 to −0.725, *P* < 0.001) than in women, indicating a more pronounced degree of vascular stiffening.

### Body Mass Index-Related Associations

Participants with a lower BMI (underweight) had significantly larger arterial ODs (β = 0.910, 95% CI: 0.158–1.661, *P* = 0.018) and IDs (β = 0.775, 95% CI: 0.082–1.469, *P* = 0.028), compared with those with normal BMI. In contrast, overweight participants showed a negative association with arterial ID (β = −0.686, 95% CI: −1.239 to −0.133, *P* = 0.015), compared with the reference group.

Venous diameters were positively associated with BMI, with overweight participants showing larger ODs (β = 0.918, 95% CI: 0.171–1.664, *P* = 0.016) and IDs (β = 0.736, 95% CI: 0.035–1.437, *P* = 0.039), compared with those with normal BMI. Arterial wall thickness also increased with BMI, with overweight participants showing a significant positive association (β = 0.198, 95% CI: 0.100–0.291, *P* < 0.001).

## Discussion

This study used OCT B-scan imaging to precisely measure retinal vessel parameters, including ODs, IDs, wall thickness, and reflectivity, which are metrics not typically captured by traditional fundus photography. Fundus photography has been widely used to estimate retinal vessel caliber, particularly through the calculation of central retinal artery and vein equivalents.^20^, ^21^, ^22^, ^23^ However, it provides a more generalized assessment, often focusing on vessel caliber without capturing the detailed structural features of vessel walls.

Muraoka et al reported weak-to-moderate correlations between vessel diameters measured by OCT and fundus photographs.^12^ Significant correlations were observed for arterial and venous diameters, with Pearson's coefficients ranging from 0.286 to 0.455.^12^ These correlations reflect inherent differences in the principles of the imaging modalities, the number of vessels evaluated, and the regions of measurement. This study did not specifically assess the correlation between OCT-based measurements and central retinal artery and vein equivalent values derived from fundus photography primarily because of the differences in measurement techniques and study designs. However, based on previous findings, we expected a moderate correlation between the measurements obtained by the 2 methods due to the similarities in measurement protocols.^12^

The focus on OCT-based metrics allows for a comprehensive assessment of the retinal vasculature and its associations with demographic and anthropometric factors such as age, sex, and BMI. These insights improve our understanding of how these factors influence retinal vessel health and suggest potential clinical applications for monitoring vascular health.

### Age-Related Changes

Most previous studies have reported a linear decline in vessel caliber with age.^24^, ^25^, ^26^, ^27^ In contrast, our findings suggest a U-shaped appearance, with arterial diameters decreasing until the 50s and increasing after that. This difference may be due to variations in the study populations, methodologies, or physiological factors. For instance, previous studies might have included populations with higher rates of hypertension or diabetes, which are conditions known to cause continuous vascular narrowing.^25^^,^^28^ In contrast, our cohort, which had a lower cardiovascular risk profile, displayed a more complex pattern. Additionally, OCT's ability to measure both IDs and ODs, as well as vessel wall characteristics, may reveal subtle changes compared with traditional methods, such as fundus photography.

Physiologically, the initial decrease in arterial diameter may reflect early vascular stiffening and endothelial dysfunction.^29^^,^^30^ The subsequent increase after 60 years of age could be a compensatory response to reduced vascular compliance, helping maintain tissue perfusion.^20^^,^^27^^,^^31^ Consistent age-related increases in arterial wall thickness and reflectivity were also observed, suggesting that vascular stiffening and collagen deposition contribute to these structural changes.^29^^,^^30^ In contrast, venous diameters showed a linear decline with age, whereas venous wall reflectivity increased to a lesser extent, indicating different aging mechanisms between arteries and veins.

### Sex Differences

In this study, women had larger arterial diameters and smaller venous diameters than male individuals, which is consistent with previous research.^21^^,^^22^^,^^29^^,^^32^^,^^33^ The differences likely arise from hormonal factors, particularly the vasodilatory effects of estrogen, which enhance endothelial function and promote vasodilation, contributing to the larger arterial diameters in female individuals.^29^^,^^33^ The smaller venous diameters in women may also reflect sex-specific vascular regulation, potentially influenced by hormonal differences, such as the effects of estrogen, which modulates vascular tone and endothelial function.^34^

Higher venous wall reflectivity in male individuals as compared to female individuals suggests more pronounced vascular stiffening, potentially due to the absence of the protective effects of estrogen and the higher prevalence of cardiovascular risk factors in men.^34^ These findings highlight the role of hormonal and vascular aging processes in shaping sex-specific differences in the retinal vasculature.^29^^,^^33^

### Body Mass Index-Related Associations

Our findings show a negative association between BMI and arterial diameters and a positive association with venous diameters, consistent with earlier studies.^35^^,^^36^ This pattern may reflect the hemodynamic stress of obesity, wherein veins dilate to accommodate increased blood volume^37^ whereas arteries constrict to maintain tissue perfusion.^38^ Additionally, a higher BMI is linked to increased arterial wall thickness, likely influenced by proinflammatory adipokines such as leptin, which impair nitric oxide-mediated vasodilation.^36^^,^^39^ These results emphasize the significant impact of obesity on the retinal vasculature, paralleling systemic vascular changes.

### OCT-Based Parameters and Clinical Significance

OCT provides consistent measurements by targeting the region around the optic disc where the major retinal vessels run parallel to the retinal plane. This study offers a nuanced understanding of retinal vascular health and its associations with key demographics and anthropometrics, such as age, sex, and BMI. Although blood pressure and hypertension are critical to vascular health, their variability due to factors such as medication, daily fluctuations, and stress led us to focus on more stable parameters, such as age, sex, and BMI, in this analysis. Future research should incorporate blood pressure and cardiovascular risk profiles to better understand their influence on retinal vessel health and enhance the predictive value of OCT-derived metrics in systemic vascular health monitoring.

### Limitations

This study has some limitations. First, although we validated the OCT-based retinal vessel measurements, direct comparisons with other imaging modalities, such as fundus photography, were not performed. Fundus photography has been useful for assessing vessel caliber; however, its inability to capture detailed vessel wall structures highlights the potential of OCT to provide a more comprehensive evaluation of vascular health. Future studies should explore how these techniques can be integrated to improve vascular assessment. Second, this study did not include clinical data, such as blood pressure, which is known to directly affect vessel caliber and wall structure. The absence of such data limited our ability to fully evaluate the relationship between systemic hypertension and retinal vessel health, highlighting the need for future studies incorporating blood pressure and other cardiovascular factors. Third, as a retrospective observational study, a multivariable analysis was used to control for confounding factors; however, unmeasured confounders may still exist. Finally, vessel branching or crossing may complicate the vascular structure, making it more difficult to obtain precise measurements. This emphasizes the need for more advanced imaging algorithms and automated tools to improve the reproducibility and precision of retinal vessel assessment.

OCT-based measurements of vessel wall thickness and reflectivity provide valuable insights into retinal vascular health. With further refinement of these metrics and the incorporation of systemic risk factors, OCT has the potential to become a valuable tool for retinal and systemic vascular health monitoring, offering the potential for future research and clinical applications.

## Data Statement

The datasets generated and analyzed in this study are available from the corresponding author upon reasonable request.

## References

[1] Wong T.Y., Mitchell P. (2007). The eye in hypertension. Lancet.

[2] Wong T.Y., Mitchell P. (2004). Hypertensive retinopathy. N Engl J Med.

[3] Parr J.C., Spears G.F. (1974). Mathematic relationships between the width of a retinal artery and the widths of its branches. Am J Ophthalmol.

[4] Parr J.C., Spears G.F. (1974). General caliber of the retinal arteries expressed as the equivalent width of the central retinal artery. Am J Ophthalmol.

[5] Hubbard L.D., Brothers R.J., King W.N. (1999). Methods for evaluation of retinal microvascular abnormalities associated with hypertension/sclerosis in the Atherosclerosis Risk in Communities Study. Ophthalmology.

[6] Sherry L.M., Wang J.J., Rochtchina E. (2002). Reliability of computer-assisted retinal vessel measurementin a population. Clin Exp Ophthalmol.

[7] Tanabe Y., Kawasaki R., Wang J.J. (2010). Retinal arteriolar narrowing predicts 5-year risk of hypertension in Japanese people: the Funagata study. Microcirculation.

[8] Kawasaki R., Xie J., Cheung N. (2012). Retinal microvascular signs and risk of stroke: the multi-ethnic study of atherosclerosis (MESA). Stroke.

[9] Seidelmann S.B., Claggett B., Bravo P.E. (2016). Retinal vessel calibers in predicting long-term cardiovascular outcomes: the atherosclerosis risk in communities study. Circulation.

[10] Polak J.F., Pencina M.J., Pencina K.M. (2011). Carotid-wall intima-media thickness and cardiovascular events. N Engl J Med.

[11] Gutierrez J., Goldman J., Dwork A.J. (2015). Brain arterial remodeling contribution to nonembolic brain infarcts in patients with HIV. Neurology.

[12] Muraoka Y., Tsujikawa A., Kumagai K. (2013). Age- and hypertension-dependent changes in retinal vessel diameter and wall thickness: an optical coherence tomography study. Am J Ophthalmol.

[13] Nakao S.Y., Miyake M., Hosoda Y. (2021). Myopia prevalence and ocular biometry features in a General Japanese population: the Nagahama study. Ophthalmology.

[14] Majithia S., Tham Y.C., Chong C.C. (2022). Retinal nerve fiber layer thickness and Rim area profiles in Asians: pooled analysis from the Asian eye epidemiology consortium. Ophthalmology.

[15] Rim T.H., Kawasaki R., Tham Y.C. (2020). Prevalence and pattern of geographic atrophy in Asia: the Asian eye epidemiology consortium. Ophthalmology.

[16] Hirota Y., Muraoka Y., Kogo T. (2023). Association of retinal pigment epithelium reflectivity on optical coherence tomography with recurrence of Vogt-Koyanagi-Harada disease: a retrospective observational study. Clin Ophthalmol.

[17] Horii T., Murakami T., Nishijima K. (2012). Relationship between fluorescein pooling and optical coherence tomographic reflectivity of cystoid spaces in diabetic macular edema. Ophthalmology.

[18] Tai E.L., Li L.J., Wan-Hazabbah W.H. (2017). Effect of axial eye length on retinal vessel parameters in 6 to 12-year-old Malay girls. PLoS One.

[19] Chang M., Yoo C., Kim S.W., Kim Y.Y. (2011). Retinal vessel diameter, retinal nerve fiber layer thickness, and intraocular pressure in Korean patients with normal-tension glaucoma. Am J Ophthalmol.

[20] Myers C.E., Klein R., Knudtson M.D. (2012). Determinants of retinal venular diameter: the beaver dam eye study. Ophthalmology.

[21] Cheung C.Y., Tay W.T., Mitchell P. (2011). Quantitative and qualitative retinal microvascular characteristics and blood pressure. J Hypertens.

[22] Yanagi M., Misumi M., Kawasaki R. (2014). Is the association between smoking and the retinal venular diameter reversible following smoking cessation?. Invest Ophthalmol Vis Sci.

[23] Brazionis L., Quinn N., Dabbah S. (2023). Review and comparison of retinal vessel calibre and geometry software and their application to diabetes, cardiovascular disease, and dementia. Graefes Arch Clin Exp Ophthalmol.

[24] Streese L., Lona G., Wagner J. (2021). Normative data and standard operating procedures for static and dynamic retinal vessel analysis as biomarker for cardiovascular risk. Sci Rep.

[25] Kaushik S., Kifley A., Mitchell P., Wang J.J. (2007). Age, blood pressure, and retinal vessel diameter: separate effects and interaction of blood pressure and age. Invest Ophthalmol Vis Sci.

[26] Klein R., Myers C.E., Knudtson M.D. (2012). Relationship of blood pressure and other factors to serial retinal arteriolar diameter measurements over time: the beaver dam eye study. Arch Ophthalmol.

[27] Michelson G., Wärntges S., Baleanu D. (2007). Morphometric age-related evaluation of small retinal vessels by scanning laser Doppler flowmetry: determination of a vessel wall index. Retina.

[28] Wei F.F., Zhang Z.Y., Thijs L. (2016). Conventional and ambulatory blood pressure as predictors of retinal arteriolar narrowing. Hypertension.

[29] Wong T.Y., Islam F.M., Klein R. (2006). Retinal vascular caliber, cardiovascular risk factors, and inflammation: the multi-ethnic study of atherosclerosis (MESA). Invest Ophthalmol Vis Sci.

[30] Kawashima-Kumagai K., Tabara Y., Yamashiro K. (2018). Association of retinal vessel calibers and longitudinal changes in arterial stiffness: the Nagahama study. J Hypertens.

[31] Ungvari Z., Tarantini S., Donato A.J. (2018). Mechanisms of vascular aging. Circ Res.

[32] Sun C., Liew G., Wang J.J. (2008). Retinal vascular caliber, blood pressure, and cardiovascular risk factors in an Asian population: the Singapore Malay Eye Study. Invest Ophthalmol Vis Sci.

[33] Wong T.Y., Klein R., Sharrett A.R. (2003). The prevalence and risk factors of retinal microvascular abnormalities in older persons: the cardiovascular Health Study. Ophthalmology.

[34] Wong T.Y., Knudtson M.D., Klein B.E. (2005). Estrogen replacement therapy and retinal vascular caliber. Ophthalmology.

[35] Wang J.J., Taylor B., Wong T.Y. (2006). Retinal vessel diameters and obesity: a population-based study in older persons. Obesity (Silver Spring).

[36] Stapleton P.A., James M.E., Goodwill A.G., Frisbee J.C. (2008). Obesity and vascular dysfunction. Pathophysiology.

[37] Magder S. (2017). Erratum to: volume and its relationship to cardiac output and venous return. Crit Care.

[38] Drew P.J., Shih A.Y., Kleinfeld D. (2011). Fluctuating and sensory-induced vasodynamics in rodent cortex extend arteriole capacity. Proc Natl Acad Sci U S A.

[39] Knudson J.D., Dincer U.D., Bratz I.N. (2007). Mechanisms of coronary dysfunction in obesity and insulin resistance. Microcirculation.

